# Techniques of assessing small airways dysfunction

**DOI:** 10.3402/ecrj.v1.25898

**Published:** 2014-10-17

**Authors:** William McNulty, Omar S. Usmani

**Affiliations:** National Heart and lung Institute, Imperial College London and Royal Brompton Hospital, London, UK

**Keywords:** chronic obstructive pulmonary disease, asthma, lung function, small airways, impulse oscillometry, multiple breath nitrogen washout, imaging

## Abstract

The small airways are defined as those less than 2 mm in diameter. They are a major site of pathology in many lung diseases, not least chronic obstructive pulmonary disease (COPD) and asthma. The small airways are frequently involved early in the course of these diseases, with significant pathology demonstrable often before the onset of symptoms or changes in spirometry and imaging. Despite their importance, they have proven relatively difficult to study. This is in part due to their relative inaccessibility to biopsy and their small size which makes their imaging difficult. Traditional lung function tests may only become abnormal once there is a significant burden of disease within them. This has led to the term ‘the quiet zone’ of the lung. In recent years, more specialised tests have been developed which may detect these changes earlier, perhaps offering the possibility of earlier diagnosis and intervention. These tests are now moving from the realms of clinical research laboratories into routine clinical practice and are increasingly useful in the diagnosis and monitoring of respiratory diseases. This article gives an overview of small airways physiology and some of the routine and more advanced tests of airway function.

The airways consist of approximately 23 generations of dichotomously branching tubes from the trachea to the alveoli (1) (Fig. 1). The main function of the airways is to ventilate the gas exchanging units of the lung. They also play a role in the conditioning of inhaled air, removal of particulate matter, and immune defence within the lung.

**Fig. 1:**
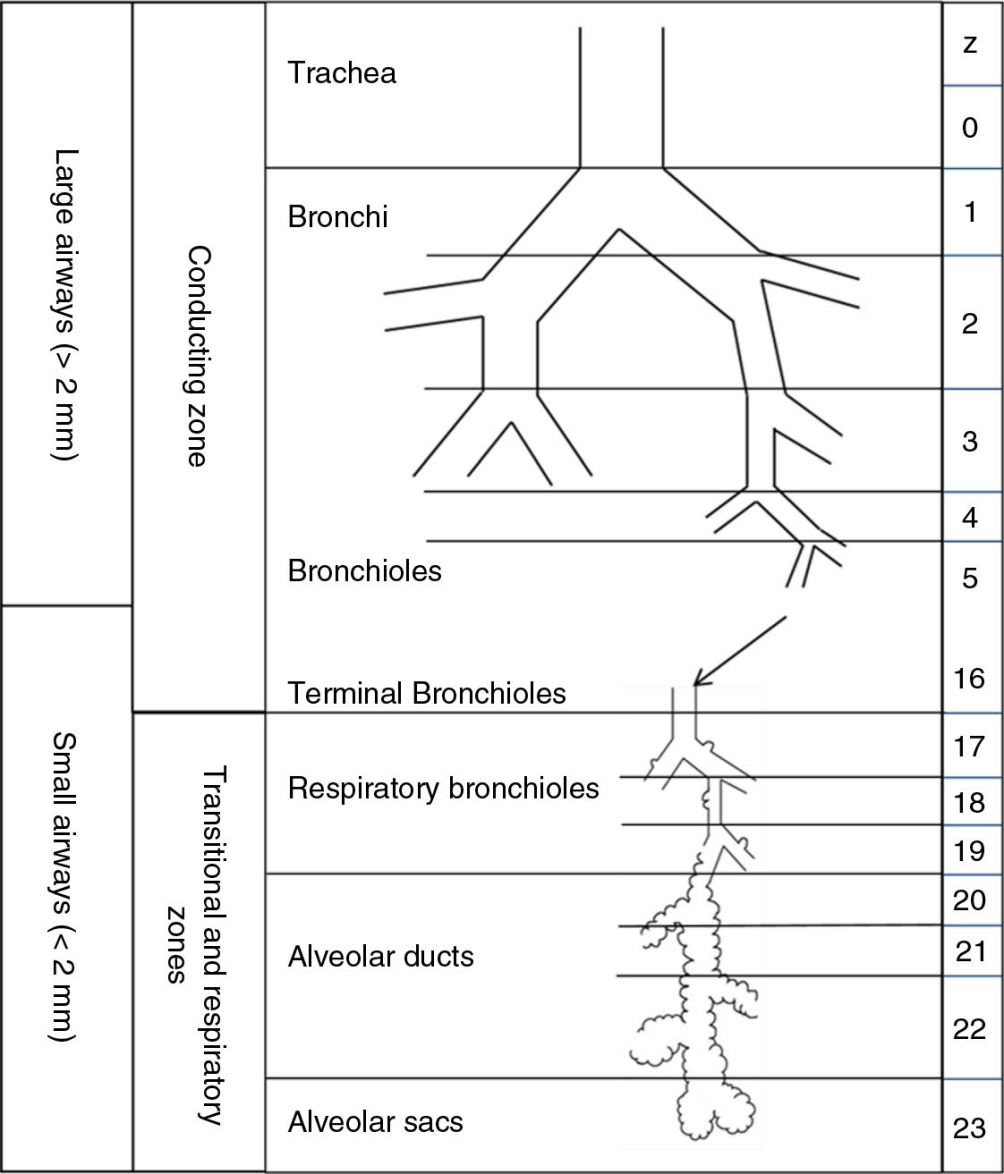
Airway generations (adapted from ref. 1).

The first 15 generations of airways are called the conducting airways and take no part in gas exchange. They constitute the anatomical dead space, which is approximately 100–150 ml in a human adult (2). Beyond this region lie the respiratory bronchioles which have occasional alveoli budding from them. These continue to divide until they reach the alveolar sacs with a total surface area of 70–80 m^2^ (3). These airways take part in gas exchange and comprise the acinar airways.

The small airways refer to those airways less than 2 mm in diameter (4). These occur from approximately generation 8 and include a portion of the conducting airways as well as all the acinar airways. They have important structural and physiological differences from large airways. First, they lack the cartilaginous support seen in large airways and lack mucous glands. They are lined by surfactant which reduces surface tension and helps prevent them from closing on expiration and at low lung volumes (5).

Throughout successive airway generations, there is a reduction in the length and diameter of the airway. Because of the exponential increase in airway numbers, there is a rapid increase in cross-sectional area with each subsequent generation. This has two major effects on airway physiology. First, for any given flow, the velocity of gas transit within the lung decreases with increasing airway generation. The result of this is high velocity flow in the proximal airways which is turbulent and hence density dependent. In the small airways of the lung, flow is laminar and therefore independent of gas density (6). At the interface of the conducting and acinar airways, there is a change from bulk convective flow to diffusion down a concentration gradient. However the distance for diffusion is small, approximately 0.2 mm (7). Second, the resistance to airflow in the small airways is low in health, comprising between 10 and 25% of total airways resistance (8, 9). However, small airways resistance is significantly increased in disease (10). Small airways resistance is largely independent of lung volume whilst large airways resistance is altered significantly with change in lung volumes (8). These arrangements in the human lung help to achieve as equitable ventilation to lung units as possible, whilst maintaining low airflow resistance and minimal work of breathing.

## The small airways in disease

Both in chronic obstructive pulmonary disease (COPD) and asthma, the small airways have been shown to be the major site of airflow obstruction (9, 11, 12). The small airways may be more prone to pathology because of their size. Small inhaled particles and pathogens may be deposited here and pathological changes in airways disease make the small airways susceptible to occlusion. Therefore, small airways may require inhaled therapeutic aerosols of smaller size to be able to penetrate the airways tree and reach the distal lung region (13). Pouseille's law states that the resistance to flow is inversely proportional to the fourth power of the radius. Hence, airway obstruction can have profound effects on lung physiology. The obstruction of small airways can occur through a number of mechanisms, including luminal occlusion by mucus, reduction in luminal diameter from inflammatory infiltrates, smooth muscle hypertrophy, or airway wall thickening. In addition, loss of structural airway supports may enhance collapsibility of airways.

### Asthma

In asthma, the small airways are thickened with a chronic inflammatory infiltrate affecting all layers of the airway (14). Inflammatory changes are present throughout the airways, although differences in the extent and composition of the inflammatory infiltrate exist between large and small airways. The small airways are the major site of inflammation in asthma (15, 16) with a chronic inflammatory infiltrate consisting of eosinophils, T-lymphocytes, neutrophils, and macrophages. In addition, there is smooth muscle thickening and luminal occlusion by mucus (17–19). In small airways, the density of the lymphocytes and eosinophils is greater in the outer walls compared to large airways where more central airway wall inflammation predominates (15, 20). Mast cells are found more commonly in the periphery of the lung (21) than the central airways and more marked neutrophilic inflammation may be seen in the peribronchiolar lung parenchyma in fatal asthma (15). The severity of inflammatory changes correlates with lung function in nocturnal asthma (22), severe asthma (21), and is more marked in patients with fatal asthma compared to non-fatal asthma (23).

### Chronic obstructive pulmonary disease

COPD is characterised predominately by neutrophilic and lymphocytic small airway infiltration along with the presence of (24–26). Lymphocytic infiltration and smooth muscle hypertrophy are more prominent in COPD than in asymptomatic smokers (25). In addition, there is airway remodelling with peribronchial fibrosis, smooth muscle hypertrophy, and luminal occlusion from mucus (27, 28). The extent of airway inflammation correlates with disease severity in COPD (24, 29, 30). However, it is airway wall thickness, rather than the severity of inflammatory changes, that is more strongly associated with disease progression in COPD (30). This suggests that regulation of the remodelling pathways through tissue growth factors may be altered in susceptible patients. Interestingly, smoking has been shown to increase tissue levels of growth factors that promote airway remodelling prior to the onset of inflammatory changes (31). Emphysematous destruction of lung tissue may also affect the small airways by disruption of the elastic fibres supporting airway walls. The extent of airway inflammation correlates with the degree of disruption (32) suggesting that peribronchiolar inflammation may drive the protease-mediated disruption of airway attachments. Indeed, small airways disease may precede emphysematous changes identified by computed tomography (CT) (33).

Inflammatory small airways disease may exacerbate small airways injury and dysfunction through mechanical stresses of cyclic opening and closing of airways during tidal breathing.

## Physiological assessment of the small airways

Small airways obstruction may lead to a reduction in airflow, increased airways resistance, gas trapping, and inhomogeneity of ventilation. Consequently, physiological tests measuring these variables can detect and quantify small airways disease (34). Table 1 summarises the techniques available for the assessment of small airways disease.

**Table 1:** Summary of physiological and imaging techniques for assessing the small airways

	Measures	Pros	Cons
Lung function			
Spirometry	FEV_1_, FEF_25–75_, FEV_1_/FVC, FEV_3_/FVC, FEV/SVC	Widely available Reproducible Standardised criteria	Relatively insensitive to early disease and subtle changes Effort dependent Not specific to small airways changes
Plethysmography	RV, RV/TLC, airways resistance	Widely available Reproducible Relatively easy to perform Sensitive to early change	Not specific for small airways disease Effort dependent Relatively time consuming
IOS	Z, R_rs_, X_rs_	Non-invasive and easy to perform Effort independent Reproducible Intra-breath analysis	Equipment not widely available Interference from swallowing and upper airway artefact
Inert gas washout	Closing capacity and closing volume Phase III slope: S_III_, S_acin_, S_cond_	Sensitive to early change Can distinguish between distal and proximal airways disease	Difficult to perform, requiring specialist equipment Restricted to research settings
Exhaled nitric oxide	FE_NO_	Easy and quick to perform Hand-held analysers available Sensitive to changes with treatment in asthma	Unclear role in COPD Affected by smoking status
Imaging			
High resolution computed tomography	Assessment of airway changes Assessment of gas trapping (MLD_E/I_)	Widely available Quick and easy to perform	Unable to visualise small airways directly Specialist software may be required No standardised measurements Radiation dose
Hyperpolarised magnetic resonance imaging	Apparent diffusion co-efficient Regional ventilation defects	Allows assessment of heterogeneity in distribution of disease No radiation dose	Expensive Limited to research applications
Nuclear medicine (scintigraphy, SPECT, and PET)	Ventilation Inhaled drug or receptor distribution	Allows assessment of heterogeneity in distribution of disease Can help target drugs to site of lung Can be tailored to study individual drugs or receptors	Radiation dose Difficult to identify small airways Some isotopes can be expensive SPECT and PET not yet widely available

FEV_1_=forced expiratory volume in 1 sec; FEV_3_=forced expiratory volume in 3 sec; FVC=forced vital capacity; R_rs_=respiratory system resistance; X_rs_=respiratory system reactance; Z=impedance; SVC=slow vital capacity; RV=residual volume; TLC=total lung capacity; FEF_25–75_=forced expiratory flow at 25–75% of vital capacity; FE_NO_=fractional expired nitric oxide; S_acin_=DCDI contribution to S_nIII_; S_cond_=CDI contribution to S_nIII_; S_III_=slope of phase III; MLD_E/I_=expiratory to inspiration mean lung density; SPECT=single photon emission computed tomography; PET=Positron emission tomography.

## Spirometry

Spirometry is the most widely used lung function test both in the diagnosis and stratification of severity of lung disease. A diagnosis of obstructive lung disease is made when the ratio of the Forced Expiratory Volume in 1 sec (FEV_1_) to Forced Vital Capacity (FVC) is less than 70% (35). Whilst a reduction in FEV_1_ may reflect airflow obstruction, it is also dependent on lung volumes, elastic recoil, respiratory muscle strength, and patient effort (36). In health, the main site of airways resistance occurs in the 4th–8th airway generations. Thus, FEV_1_ largely reflects large airways obstruction, and a significant amount of small airways disease must accumulate before FEV_1_ becomes abnormal.

Examination of the mid-portion of expiratory flow may offer more information on small airway pathology. The Forced Expiratory Flow between 25 and 75% of the FVC (FEF_25–75_) is one of the most commonly cited measures of small airways pathology. McFadden and Linden postulated that the latter part of the vital capacity was affected by increased resistance in small airways as lung volume fell. Pathology in these airways causes excessive airway narrowing and collapse at an earlier time and closer to the alveolus during exhalation. This results in a reduction in the maximum expiratory flow that can be achieved (37). However, FEF_25–75_ is dependent on the FVC and therefore changes in FVC will affect the portion of the flow-volume curve examined. If FEF_25–75_ is not adjusted for lung volume, there is poor reproducibility (38). Another disadvantage is the sensitivity of the FEF_25–75_, as it is frequently normal if the FEV_1_/FVC ratio is >75% (39). In addition, there is poor correlation with other markers of small airways disease such as gas trapping (40) and histological evidence of small airways inflammation (41). The Forced Expiratory Volume in 3 sec (FEV_3_) to FVC ratio has been suggested as an alternative measure of small airways disease. The fraction of air not expired in the first 3 sec (1-FEV_3_/FVC) is also calculated to estimate the growing proportion of long time constant lung units. As FEV_1_/FVC falls, the FEV_3_/FVC falls and the 1-FEV_3_/FVC rises. These measures have a better accuracy than FEF_25–75_, particularly in advancing age (42).

Gibbons et al. (43) suggested that the change in FVC following a histamine provocation is a better measure of small airway dysfunction in asthmatic patients than the fall in FEV_1_. A fall in FVC suggests small airway closure and gas trapping. Other spirometric markers that have been suggessted for assessment of small airways disease have included the ratio of the FVC to slow vital capacity (SVC) (44).

## Plethysmography

Plethysmographic assessment of lung volumes provides a sensitive measure of gas trapping and lung hyperinflation. Hyperinflation may be defined as an abnormal elevation of lung volumes at the end of expiration (45). It is a function of airflow limitation, lung elastic recoil, and chest wall compliance. Airway narrowing results in a prolonged time constant for expiration, and airways may close resulting in gas trapping. The residual volume (RV) is an important measure of small airways dysfunction and may be raised before the onset of abnormal spirometry in asthma (46, 47). The RV correlates with the degree of inflammatory changes in small airways in COPD (24) and with peripheral airway resistance in asthma (48). Indeed, improvement in asthma symptoms following treatment with monteleukast correlated with the reduction in RV but not spirometric parameters (49).

The residual volume/total lung capacity (RV/TLC) ratio may be a more useful marker of gas trapping as the TLC is frequently raised in obstructive lung disease. Sorkness et al. demonstrated that the RV/TLC ratio is higher in patients with severe asthma compared to non-severe asthma and correlates inversely with FVC (40). However, the upper limit of normal value varies with age and sex and therefore the predicted value may provide a better measure of gas trapping than the absolute value.

Airways resistance (*R*_*aw*_) may also be measured by assessing pressure and flow at the mouth during body plethysmography. Airways resistance is increased in obstructive lung diseases and is more sensitive to changes than spirometry in detecting bronchodilation (50). However, it is not specific for the small airways which limits its application in diagnosing and monitoring distal airways disease (51).

## Impulse oscillometry

Impulse oscillometry (IOS) applies oscillating pressure variations in the form of random noise to the respiratory system in order to determine the mechanical properties of the lung. The multiple frequencies between 3 and 20 Hz are applied over normal tidal breathing from a loudspeaker. The resulting pressure and flow changes are measured at the mouth and analysed in a Fourier transformation to determine the impedance (Z) of the respiratory system. This is composed of the in-phase or ‘real’ part of the impedance, known as resistance (R_rs_), and the out of phase or ‘imaginary’ part, called reactance (X_rs_). In health, R_rs_ is independent of oscillation frequency but becomes frequency dependent in the presence of airways obstruction. Reactance is determined by the elastic and the inertial properties of the lung and is frequency dependent. At low frequencies, X_rs_ is negative and largely represents the elastic forces within the lung. At high frequencies, X_rs_ is positive and is determined by inertiance within the lung resulting from acceleration of airflow. At a point where the elastance and inertiance are equal and opposite, X_rs_ is 0. This is known as the resonant frequency (F_res_) and occurs between 8 and 12 Hz in healthy patients (52).

Higher frequency signals (>15 Hz) are absorbed by the respiratory system before reaching the small airways and hence reflect the contribution of large airways. Low frequencies (5 Hz) penetrate deep into the lung and therefore represent the whole lung. The contribution of the distal airways may be determined by the difference between R5 and R20, and therefore can give insight into small airways pathology. However, the anatomical location of the transition between the small and large airways has not been determined (53). Despite this, there is evidence that low frequency resistance and reactance measurements correlate strongly with transpulmonary resistance measured by oesophageal manometry (54) and other traditional small airways measures (55).

When airway obstruction is present, R_rs_ becomes frequency dependent with a predominant increase in low frequency resistance. This has been shown to identify patients with asthma (56–59) and COPD (56, 60, 61). Whilst R_rs_ does increase in early stage COPD (60), reactance measures are better at identifying severity of disease (62) and are more closely associated with other parameters including FEV_1_ and measures of hyperinflation (61). Dyspnoea scores and health status correlate significantly with R_5–20_ and X_5_ quality of life in stable COPD and are sensitive to improvements following exacerbations (63).

IOS also allows for the discrimination of inspiratory and expiratory resistance and reactance. Inspiratory minus expiratory reactance at 5 Hz (ΔX_5_) has been shown to help discriminate between asthma and COPD (64). In addition, it has also been shown to be a sensitive, non-invasive method of detecting expiratory flow limitation (EFL) in COPD. Expiratory reactance falls when EFL is present as the pressure signals cannot pass the choke point with in the airway (65, 66). This is likely to be due to the enhanced collapsibility of airways in expiration and is a major factor in the development of dynamic hyperinflation. Indeed, recent studies using R_5–20_as an index of distal airway abnormality have shown the presence of small airways dysfunction even in patients with mild-moderate asthma (67).

Studies examining the effect of inhaled therapies on lung mechanics have demonstrated that IOS is sensitive to bronchodilation in both COPD (50, 68) and asthma (69, 70). It has also been used in the assessment of lung transplant recipients for bronchiolitis obliterans (71) and following environmental exposure to dusts (72, 73).

IOS has the advantage of being simple to use and is effort independent. It provides continuous measurement of pulmonary mechanics giving a high temporal resolution allowing intra-breath analysis. As IOS does not rely on forced manoeuvres, it may be more suitable for patients who cannot perform these easily such as children or those with severe lung diseases. This may also reduce the effects of premature airway closure seen during forced spirometry manoeuvres. Interference from upper airways artefacts such as tongue movement or swallowing can make assessment difficult. Patients undergoing IOS do need some coaching for accurate measures to be made.

## Inert gas washout

Gas washout techniques were introduced in the 1950s as a way of measuring the efficiency of gas mixing within the lungs. This is dependent on the structure of both the large and small airways and hence information regarding these can be inferred from the tests. The most commonly employed technique is the single breath nitrogen washout (SBNW) and more recently the multiple breath nitrogen washout (MBNW). Other gases may be used including helium and sulphur hexafluoride (SF_6_) whose physical properties determine gas flow within the lung.

## Single breath nitrogen washout

The SBNW is performed by inhaling 100% oxygen from RV to TLC followed by a SVC exhalation. The exhaled volume and nitrogen concentration is measured and the resulting trace can be broken down into four distinct phases.

In phase I, the nitrogen concentration is close to 0% as this represents anatomical dead space where there is no gas mixing. During phase II, there is a sharp rise in the expired nitrogen concentration as dead space gas mixes with resident alveolar gas. Phase III represents alveolar gas and the expired nitrogen concentration begins to plateau, although there is a slight rise from the start to finish of this phase due to ventilation heterogeneity. This occurs whenever two lung units are ventilated to a different degree and the best ventilated unit will empty preferentially before a less well-ventilated lung unit. In health, this occurs to a degree because of asymmetry in lung structure and due to the effects of gravity on the base of the lung resulting in longer time constants for emptying. Finally, in phase IV, there is a steep rise in expired N_2_ concentration as the most poorly ventilated areas (with little O_2_ mixing) empty. This is also the point at which the small airways start to close as a result of gravity-dependent collapse and is known as the closing volume (CV). The CV and RV together are known as the closing capacity (CC). Normally, small airways closure occurs close to RV. However, small airways disease may cause premature airway collapse resulting in an increased CV and gas trapping. CV may be expressed as a ratio of VC and should not exceed 25% (74). The CC may be expressed as a ratio of TLC and is useful in obstructive lung diseases.

Analysis of the slope of phase III (S_III_) provides information on the ventilation heterogeneity in the lung. Airways diseases do not affect the lung uniformly and this results in disparities in the ventilation of individual subunits. This may occur in the conducting airways where gas flows by convection (convection-dependent ventilation inhomogeneity, CDI) and results from narrowing of airways or increased stiffness in the subtended lung units. It may also occur in the very distal acinar airways where the diffusion–convection front arises (diffusion–convection-dependent inhomogeneity, DCDI). Here, it occurs as a result of structural asymmetry between lung units (75). Thus, where airways disease occurs, those affected lung units mix less well with the inspired oxygen (and thus have a higher nitrogen concentration) and empty more slowly. This causes an increase in S_III_.

SBNW indices have been used in the assessment and response to treatment in both asthma and COPD. Asthmatic patients with a normal FEV_1_ have increased CV and phase III slope compared to healthy controls. In addition, the frequency of exacerbations correlates with S_III_ suggesting it may be a sensitive measure of patients with poor control (76). Indeed, increased CV in patients with severe asthma has been shown to be a risk factor for predicting an exacerbation (77). Levels of exhaled markers of airway inflammation including nitric oxide correlate with S_III_ and CC/TLC ratio in asthma (78, 79). Furthermore, severe, steroid-dependent asthmatic patients have more marked changes in SBNW indices than patients with mild to moderate asthma (79). These markers have also been used to assess changes following both inhaled and oral therapies for asthma (80–83).

Over 35 years ago, the S_III_ of the SBNW was recognised as being more closely related to histological small airways inflammation in COPD than FEF_25–75_ (29). Further evidence of its association with small airways inflammation in COPD came from the examination of bronchial biopsies and bronchoalveolar lavage (BAL) specimens (84). COPD severity may also be predicted by changes in SBNW indices as the S_III_ correlates with FEV_1_ (85) and TL_CO_ in alpha-1 antitrypsin deficiency (86).

SBNW is sensitive to early changes in airways in smokers with an increase in CV (87), but its use is controversial in COPD. Buist et al. demonstrated that many smokers with normal spirometry, but abnormal small airway indices, did not go on to develop obstructive spirometry over a 9–11 year follow-up. However, of those that did, the CC/TLC ratio predicted the rate of decline in FEV_1_ suggesting it may be useful in identifying at risk smokers (88). Stănescu et al. similarly found that in a group of smokers and ex-smokers with normal spirometry, over half had abnormal small airway indices, yet most still had normal spirometry 13 years later. In their cohort, a high S_III_ predicted accelerated decline in FEV_1_ (89).

Despite its sensitivity, the SBNW is not specific to small airways pathology. Changes in any of the generations of the conducting airways will also affect the slope of phase III. Thus, whilst it is possible to infer that a normal S_III_ indicates no small airways disease, the test is unable to locate the anatomical site of the pathology (34).

## Multiple breath nitrogen washout

**Fig. 2:**
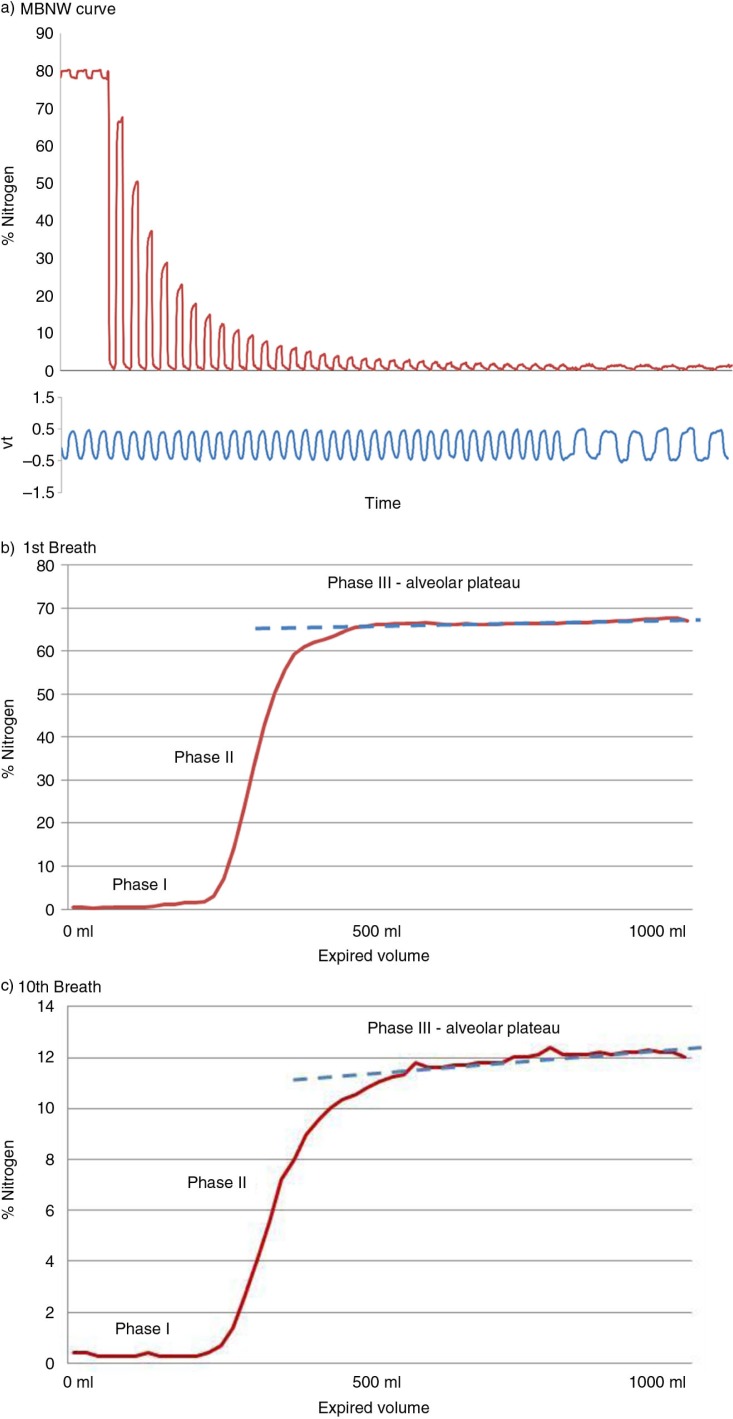
(a) Multiple breath nitrogen washout curve with individual breaths demonstrating Phase III slope (SnIII) from 1st (b) and 10th (c) breaths.

The MBNW is a modification of the single breath technique. The patient inhales 100% O_2_ from FRC with a fixed tidal volume and respiratory rate to wash out the resident nitrogen from the lungs. The test continues until the exhaled nitrogen is less than 1/40th of the original concentration (approximately 2%) for three successive breaths. The speed and efficiency of gas mixing is determined by tidal volume, breath frequency, and ventilation heterogeneity. Thus, by keeping breath frequency and tidal volume relatively constant, inferences about ventilation heterogeneity can be made (90). Figure 2 demonstrates the nitrogen washout curves from a MBNW test.

This technique allows for measurement of the efficiency of gas mixing in the whole lung through the lung clearance index (LCI). It is defined as the number of lung turnovers (FRC equivalents) required to wash out the tracer gas to 1/40th of the original concentration. This is calculated by measuring the cumulative expired volume (CEV) required to washout the resident nitrogen and dividing it by FRC:

$$\text{LCI}=\frac{\text{CEV}}{\text{FRC}}$$

FRC may be calculated during the MBNW from the following formula, whereby the volume of tracer gas (i.e. N_2_) is divided by the end-tidal concentration of the tracer gas in the first breath minus the end-tidal concentration of the tracer gas in the last breath:

$$\text{FRC}=\frac{{\text{V}}_{\left[\text{tracer}\right]}}{{\text{C}}_{\mathrm{int}}-{\text{C}}_{\text{end}}}$$

The LCI has been used extensively as a measure of airways function in cystic fibrosis and asthma, particularly in the paediatric population (91).

As a MBNW progresses, the S_III_ of each breath changes throughout the test, becoming steeper with successive breaths. In order to compare breaths within a test, the slopes must be normalised for the mean expired nitrogen concentration for each breath (S_nIII_). In normal lungs, the DCDI is the major determinant of the S_nIII_ and reaches its maximum at approximately 1.5 lung turnovers. After this, the increase in S_nIII_ is diffusion independent and hence reflects CDI (92). This allows for the quantification of the contribution of the CDI component, referred to as S_cond_, and the DCDI component, referred to as S_acin_ (75). Thus, these indices have the ability to anatomically locate the site of the airway pathology that result in ventilation inhomogeneity.

These indices have proven very sensitive, becoming abnormal in smokers with more than a 10-year pack history. In contrast, spirometric abnormalities only become abnormal after a 20 pack-year history of smoking. For smokers with *a* > 30 pack-year history and TL_CO_<60% predicted, there were proportionately larger changes in S_acin_ than S_cond_, reflecting parenchymal destruction (93). Smokers without COPD who were able to stop smoking showed sustained reversibility in S_cond_ (94). This supports the hypothesis that the major site of pathology in smoking-related lung disease starts in the peripheral airways.

These abnormalities have been further described in both asthma and COPD. Verbanck et al. demonstrated in COPD patients that both S_cond_ and S_acin_ are raised yet reflect different pathologies. S_cond_ correlated with airways measures such as FEV_1_ and specific airways resistance whilst S_acin_ was more closely associated with diffusing capacity (95). Asthmatic patients also have raised S_cond_ and S_acin_, although acinar ventilation heterogeneity is less pronounced than in COPD, presumably reflecting the degree of parenchymal destruction in COPD. In addition, asthmatic patients demonstrated bronchodilator reversibility in both S_acin_ and S_cond_, whilst COPD patients did not (96). In asthma, S_acin_ is more closely associated with airway inflammation (97) and severity in unstable patients (98). It has recently been shown that measures of ventilation heterogeneity are associated with levels of asthma control and may also predict the response to inhaled therapy (99, 100). With their sensitivity to small airways disease, they have been used in a variety of research settings. These include the assessment of inhaled treatments in both asthma (101, 102) and COPD (103), assessment of airway hyper-responsiveness (104, 105), and monitoring of lung transplant recipients (106). However, they are not yet used in routine clinical practice as there are few commercially available machines, and interpretation of results can be difficult. Theoretically, abnormalities in any of the conducting airways from the first generation can cause abnormalities in S_cond_ and therefore it is not specific to small airways. Interpreting the results with information from spirometry will help clarify this. In addition, theoretical modelling for localisation of airways disease was performed in normal subjects. It is possible that the convection–diffusion front is different in disease states and hence anatomical localisations may not be precise.

## Helium and Sulphur hexafluoride washout tests

Other inert gasses including helium and SF_6_ may be used in small concentrations as tracer gasses. These require a wash-in period and specialised analytical equipment. However, they have the added benefit that the physiochemical properties can be exploited to gain further information from the S_III_. The diffusion front of helium lies more proximally than SF_6_ and therefore changes in the helium S_III_ compared to SF_6_ S_III_ suggest more proximal acinar changes. Where both S_III_ change so that the difference between them is still the same, the possibilities are either a change in the conducting airways or concomitant effects in the proximal and distal parts of the acinus (34). There are fewer clinical studies reporting SF6 as a tracer gas and these have largely been performed in children with cystic fibrosis (107–109).

## Exhaled nitric oxide

Nitric oxide is produced in both the resident airway cells and the inflammatory cells in the lung and has a role in the regulation of airway function. Fractional exhaled nitric oxide (FE_NO_) may be measured in a single exhalation during tidal breathing. It reflects levels of inflammation, particularly eosinophillic inflammation, within the lung (110). Exhaled nitric oxide (eNO) exhibits flow rate dependency, with an inverse correlation between flow rate and FE_NO_ (111). This reflects both the transit time of exhaled gas and diffusion from the tissue as well as the compartment of the lung from which the NO was produced. Under low flow conditions, FE_NO_ largely reflects central airways and at higher flows it represents alveolar NO (112–114). This may help to localise the site of inflammation within the lung. Indeed, Lehtimäki et al. demonstrated that patients with alveolitis had higher levels of alveolar NO than asthmatic patients, who in turn have higher bronchial NO. In patients with alveolitis, alveolar NO correlated with transfer factor and alveolar volume, whilst bronchial NO correlated with airways’ hyper-responsiveness in asthmatic patients. Both groups of patients showed an improvement in FE_NO_ with steroid treatment, suggesting it is responsive to intervention (115). However, back-diffusion of NO between the alveolar and airway compartments complicates the interpretation of results. It has been recognised that NO will diffuse from the airways down a concentration gradient into the alveoli, thus elevating alveolar NO and reducing measured airway NO (116, 117). Models to correct this have been developed, however, in disease states where airways are narrowed or occluded; less NO can back-diffuse, resulting in higher FE_NO_ and lower alveolar concentrations (118). It should also be noted that current smoking reduces FE_NO_ levels and thus the smoking status of a patient needs to be taken into account when interpreting results (110).

FE_NO_ has been used extensively in asthma clinical research and practice. Central airways appear to be the major sites of production of NO in asthma both in stable populations and during exacerbations (119). Alveolar NO concentrations are raised in severe asthmatics where they correlate with alveolar eosinophillic inflammation (120) and other measures of small airways dysfunction (121). Recently, it has also been shown that alveolar NO is also raised in patients with mild asthma (122). FE_NO_ is improved by both oral (123, (124)) and inhaled corticosteroids (ICS) (125) and a raised FENO level before ICS treatment predicts an improvement in asthma control (126). This has made FE_NO_ an attractive prospect for adding to asthma treatment algorithms. However, the results of studies assessing impact of measuring FE_NO_ have been mixed. Meta-analyses suggest no overall benefit to asthma control and quality of life, but there is a reduction in ICS use in adults although an increase in ICS use in children (127, 128).

The role of FE_NO_ in COPD is less clear. FE_NO_ may be raised in COPD (129–131), although it is lower compared to asthmatic patients. An inverse correlation with FEV_1_, transfer factor, and oxygen saturations has been reported (129). Contrary to this, Gelb et al. found no difference in baseline alveolar or airway NO levels between healthy controls and aged-matched COPD patients. Despite this, the addition of salmeterol 50 mcg/fluticasone 250 mcg combination inhaler significantly reduced airway, but not alveolar NO. There was no correlation between emphysema score and exhaled NO parameters (119). Higher FE_NO_ levels may help predict a clinical response to ICS as assessed by FEV_1_ reversibility (132) and this is associated with a higher sputum eosinophil count (133).

## Imaging of the small airways

Imaging already plays an extensive role in the management of airways disease and can be used as a non-invasive measure of small airways function. Where global measures of lung function such as spirometry may classify patients of the same severity, imaging is useful in separating different phenotypes and localising heterogeneity. However, direct measurement of small airways is difficult as they are largely beyond the resolution of CT and MRI scanners. Nevertheless, both large airways have been assessed directly and the smaller airways by their impact on gas trapping and ventilation distribution. This provides both anatomical and functional information to the physician.

## High resolution CT

The small airways are beyond the resolution of CT scanners and difficult to assess directly (134). Airways as small as 2–2.5 mm in diameter can be visualised. McDonough et al. found fewer of these airways in patients with COPD undergoing CT lung cancer screening. The reduction in airway number worsened as COPD severity increased by stage, consistent with pathological findings in lung specimens (135). However, the accuracy of measurement of smaller airways may be problematic due to measurement error and artefact from breathing or cardiogenic oscillations. Nakano et al. demonstrated that measurement of intermediate-sized airways could predict the small airway dimensions measured by histology (136); thus, assessment may still prove useful in estimating the extent of small airways disease. Quantitative assessment of more proximal airway luminal diameter and airway wall thickening measured by CT correlate with lung function in COPD (137–140), with the strength of correlation increasing for more distal airways (139).

Small airways disease results in gas trapping and may be seen as areas of low attenuation distal to the site of obstruction. Mosaic attenuation reflects localised areas of gas trapping and suggests heterogeneous distribution of airways disease. It may be seen in both asthma (141) and COPD (142). However, gas trapping is best assessed on expiratory scans and may provide an indirect measure of small airways function (143). Comparing the mean lung density between expiratory and inspiratory CT provides a quantitative measure called MLD_E/I_. In asthma, MLD_E/I_ correlates strongly with FEV_1_, FEV_1_/FVC ratio, FEF_25–75_, and RV/TLC, suggesting that it reflects small airways disease (144). In children, gas trapping has been shown to be associated with improvements in post-bronchodilator R_5_ and X_5_ measured by IOS (145). Gas trapping is more marked during acute exacerbations of asthma and shows responsiveness to steroids (146). Asthmatic patients with gas trapping are more likely to have had asthma-related hospital admissions, intensive care treatment, high levels of airway neutrophils, and more severe airflow obstruction than those without (147). ICS have been shown to improve gas trapping in asthma, although in these small studies no significant benefit in lung function or spirometry was seen (148, 149).

The assessment of gas trapping in COPD is more complicated as both small airways disease and emphysema give rise to low attenuation areas. Visual estimation of emphysema provides better correlation with lung function measures such as TL_CO_, whereas MLD_E/I_ correlates well with lung function measures of gas trapping (150). In a large cohort of patients in the COPDGene study, expiratory scans with a threshold of −856 Hounsfield units (HU) for assessment of gas trapping had a stronger correlation with airflow obstruction than emphysema scores measured by the area of lung with −950 HU on inspiratory scans. The volume change between inspiratory and expiratory scans also reduced as COPD severity increased, reflecting more severe airway obstruction and gas trapping (151). Using MLD_E/I_ scores to assess gas trapping and the 15th percentile of lung density to assess emphysema, Hartley et al. demonstrated that small airways disease contributes more strongly than emphysema to severity of COPD (152). MLD_E/I_ also correlates with inflammatory changes measured by sputum neutrophils, adding further support to the inflammatory nature of small airways disease in COPD. MLD_E/I_ is sensitive to early small airways changes and correlates with S_III_ in SBNW in a group of asymptomatic, non-smokers (153). However, MLDE/I can show considerable variation between scans in individual patients and therefore may be difficult to use as a marker of response to treatment. Recently, in the research arena, static images obtained with CT have been made more functional by means of computational fluid dynamics (154) and biomarkers based on CT imaging have been developed that allow an assessment of functional small airways disease (155).

Whilst CT is a useful, non-invasive tool for indirectly assessing small airways function, it has a number of limitations. The exposure to radiation means that repeated assessment for monitoring is not feasible. There is no standardised measure of gas trapping at present and different authors have used different density thresholds for assessing gas trapping, making comparison more difficult. Gas trapping is not diagnostic for specific airways diseases and patterns such as mosaic attenuation are also seen in pulmonary vascular disease (156).

## Hyperpolarised helium magnetic resonance imaging

Hyperpolarised helium magnetic resonance imaging (^3^He MRI) allows for the assessment of distribution of ventilation and morphometry of the distal airways and lung parenchyma without exposure to ionising radiation (157). Diffusion imaging visualises the movement of ^3^He in the peripheral airspaces, bound by alveolar and airways walls. This is calculated as the apparent diffusion co-efficient (ADC) and gives insight into the microstructure of the distal airspaces (158). ADC is increased in healthy smokers with normal lung function and correlates with smoking history (159, 160), suggesting it is a sensitive marker of early damage. It is increased further in COPD where it correlates with lung function (161) and emphysematous destruction (162). Indeed, ADC correlates well with CT-derived emphysema scores and more strongly with TL_CO_ than CT-derived emphysema scores (163). In COPD patients observed over 26 months, ADC and other parameters derived from ^3^He MRI have been shown to decline whilst FEV_1_ remained stable, suggesting it is also sensitive to change over time (164). Measuring diffusion over longer periods allows the assessment of collateral ventilation in emphysema (165, 166).

Quantification of regional ventilation can be achieved by both static and dynamic assessment of ^3^He distribution within the lung. Ventilation defects are present in asthma (167, 168) and COPD (169, 170), resulting from airway narrowing or obstruction and uneven ventilation. In a group of asthmatic patients, the areas of ventilation defects were persistent or recurred in the same locations over time (169). Inflammatory cells obtained at bronchoalveolar lavage were more numerous in lobes with higher ventilation defects that those without, suggesting that the defects are the result of inflammatory airway narrowing (171). Dynamic ventilation is a more recent advance that allows imaging and assessment of ventilation with a high spatial and temporal resolution over the course of a respiratory cycle (161, 172). In asthma, areas of differential gas clearance have been observed that corroborat with evidence of airflow obstruction and gas trapping on CT (173).

Hyperpolarised MRI has the ability to assess regional lung function which makes it a useful tool in assessing airways diseases which have a heterogeneous distribution. However, the technique is still largely restricted to research applications and its role in the clinical management of airways disease is not yet clear.

## Nuclear medicine techniques

### Two-dimensional gamma scintigraphy

Two-dimensional (2-D) gamma scintigraphy has been in use for several decades. Gamma-emitting radionucleides deposited within the lung can be imaged as they decay. This allows for an assessment of the overall lung deposition and to some extent, regional differences in deposition. Incorporating radionucleide into drug compounds is challenging and hence an isotope bound to the drug such as ^99m^Tc is more commonly used. These techniques must be validated to ensure that the addition of a radiolabel does not significantly change the behaviour of the drug (174).

2-D gamma scintigraphy has been used to assess the effect of particle size on deposition within the lungs. Usmani et al. studied three particle sizes of radiolabelled salbutamol and found that whilst small particle (1.5 micron) salbutamol was associated with a higher total lung dose and more peripheral deposition, it was the large particles (6 micron) deposited in the more proximal airways that had the greatest effect on bronchodilation (13). In addition, the effect of late inhalation of a dry powder demonstrated that a higher proportion reaches the periphery of the lung without a change in the total lung dose (175).

Whilst scintigraphy does involve exposure to ionising radiation, the dose is low and is estimated at 0.15 mSv per study (176). However, 2-D imaging does not allow precise localisation of drug deposition as both central and small airways as well as alveolar distribution may contribute to gamma counts for any given area. Whilst assessment of deposition is a useful marker of drug distribution, it does not itself provide an assessment of clinical or physiological response. Hence, these studies must be assessed along with clinical and physiological data in order to evaluate efficacy.

### Single photon emission computed tomography

Single photon emission computed tomography (SPECT) is a 3-D imaging modality using multiple gamma detectors that rotate around a supine patient. Reconstruction of the images can demonstrate the radionucleide distribution in three dimensions, thereby offering superior assessment of regional lung ventilation or particle distribution. SPECT may be combined with X-ray CT to relate the radionuclide distribution to anatomical information (177, 178). SPECT can be used to image ventilation using either radiolabelled gasses or ultrafine particles such as Technegas^®^. This is an ultrafine carbon particle labelled with ^99m^Tc that has been shown to have a similar inhaled distribution in healthy patients to gases (179). This allows measurement of the extent of regional distribution of airflow. In healthy patients, airway closure measured with SPECT correlates with CC measured by SBNW. However, in asthmatic patients this correlation is lost, possibly due to regional heterogeneity in airway closure (180). Technegas SPECT has also been shown to identify regional EFL in asthma even when flow measurements or negative expiratory pressure techniques are insensitive to it (181). In COPD, Technegas SPECT can identify regional differences in emphysema which correlates with lung function and emphysema scores (182, 183). The technique can be combined with perfusion imaging to assess ventilation–perfusion relationships in the lung (184).

SPECT has proven a useful tool in defining the deposition of inhaled drugs and can allow for treatments to be more specifically targeted to areas of the lung. Ciclesonide has been shown to have good peripheral lung deposition, with low oropharyngeal deposition in both health (185) and asthma (186). Limitations of SPECT scanning include higher radiation doses to patients and a longer acquisition time. This limits the assessment of deposition of molecules with a fast clearance. However, fast SPECT protocols have been developed with image acquisition times under 1 min. This allows for assessment of both deposition and clearance of tracers (187).

### Positron emission tomography

Positron emission tomography (PET) is an emerging technique for assessment of airways disease. It can be used to assess drug deposition (188), inflammation (189), and ventilation perfusion relationships in the lung (190). However, PET scanners and the facilities to produce radioisotopes are expensive. The radioisotopes used often have a short half-life and incorporating them into drugs is a complex process. However, it has the ability to produce higher resolution images than SPECT and allows for targeting of radioisotopes to specific receptors and targets within the lung. Therefore, it is likely to be an area of exciting research in the assessment of small airways disease and treatment (191).

## Conclusions

An understanding of the role of small airways in COPD and asthma is increasingly important as it becomes necessary to distinguish individual phenotypes of the diseases. This will lead to a more tailored approach to assessment and treatment of patients with the aim of improving symptoms and function (192). It may also allow us to reduce unnecessary exposure to treatments that carry significant side effects. Given the anatomical, functional, and physiological information that can be obtained from these different tests, it is likely that a combination of investigations will be required to give the clearest picture of an individual's phenotype. At present, however, many of these investigations remain in the realm of the research laboratory and further work is required to understand their significance and interpretation in the management of these diseases.
